# The Role of Oxytocin and Sex in Analgesic Placebo-Response: Exploratory Analysis from a Sham Randomized Clinical Trial in Chronic Back-Pain Patients †

**DOI:** 10.3390/jcm14207348

**Published:** 2025-10-17

**Authors:** Rinat Mendelson-Keypur, Adi Shani, Michal Granot, Mariana Ribolhos Agostinho, Eilam Paltzur, Roi Treister, Nimrod Rahamimov

**Affiliations:** 1 Department of Psychology, University of Haifa, Mount Carmel, Haifa 3498838, Israel; rinat.mn1@gmail.com; 2 The Cheryl Spencer Department of Nursing, Faculty of Social Welfare and Health Sciences, University of Haifa, Mount Carmel, Haifa 3498838, Israel; a.eilat888@gmail.com (A.S.); mgranot@univ.haifa.ac.il (M.G.); mariana.ribolhos6@gmail.com (M.R.A.); rtreister@univ.haifa.ac.il (R.T.); 3 Department of Orthopedics B and Spine Surgery, Galilee Medical Center, Nahariya 2222605, Israel; 4 Oncologic Day Care Unit, Galilee Medical Center, Nahariya 2222605, Israel; 5 Centre for Interdisciplinary Health Research (CIIS), Faculty of Health Sciences and Nursing, Universidade Católica Portuguesa, 1649-023 Lisbon, Portugal; 6 Faculty of Medicine, Bar Ilan Medical School, Tsfat 1322456, Israel; eilamp@gmc.gov.il

**Keywords:** placebo analgesia, placebo response, biological sex, chronic back pain, oxytocin

## Abstract

**Background:** Several studies suggest that exogenous oxytocin nasal spray may enhance placebo analgesia in healthy volunteers and experimental pain models, although the findings remain mixed. The oxytocin placebo hypothesis suggests that increased oxytocin levels trigger a cascade of brain processes that boost positive expectations and augment the placebo response. Since endogenous oxytocin secretion has been found to increase during positive interactions, we hypothesized that changes in endogenous oxytocin levels will affect placebo analgesia in chronic-back-pain patients. Given the role sex has in both placebo analgesia and oxytocin secretion, we hypothesized that the response magnitude will differ by sex. **Methods:** Chronic-back-pain patients (*n* = 112) were prospectively recruited and received placebo injections. The placebo response was calculated as the change in the back-pain Visual Analog Score (VAS), and changes between pre- and post-injection salivary oxytocin levels were measured. The effect of sex and changes in oxytocin levels on pain reduction was calculated using two-way analysis of variance (ANCOVA). **Results:** Oxytocin levels decreased in 62.5% of participants and increased in 37.5%. Increased oxytocin levels were associated with greater pain reduction than decreased oxytocin levels (*p* = 0.024). Females exhibited greater pain reduction than males (*p* = 0.034). No interaction between the oxytocin change pattern and sex was observed. **Conclusions:** This study demonstrates that following a placebo injection, patients suffering from chronic back pain, who exhibited an increase in endogenous oxytocin levels, showed a higher placebo response. Females had a greater placebo response, but this was not associated with an endogenous oxytocin change. These results provide initial support for the oxytocin placebo hypothesis.

## 1. Introduction

Chronic back pain, defined as an episode of pain in the axial spine lasting more than three months, is estimated to affect approximately 20% of the population worldwide [1], is linked with substantial disability, and negatively affects social and professional function [2,3,4]. In the absence of a clear etiology, treatment primarily aims to alleviate pain while minimizing the risk of adverse effects from pharmaceutical or interventional approaches [5,6].

The much-studied analgesic placebo response, reflecting pain alleviation following placebo treatment [7,8,9,10,11,12], consists of the placebo effect (namely the therapeutic effect of the contextual components of the treatment, highly affected by expectations and learning), and other non-specific factors such as natural history, regression to the mean, or spontaneous remission [13]. Among the various factors that contribute to the placebo effect, therapeutic interactions involving warmth, empathy, trust, and competence are considered facilitators of the placebo response [14,15,16,17].

A potential factor in this response is oxytocin [18], a polypeptide synthesized in the hypothalamus and secreted by the posterior pituitary [19] which has been extensively studied for its role in various social contexts such as social bonding and interpersonal connection [20]. Peripheral blood oxytocin fluctuations pre- and post-social-interactions might be considered a biomarker for social-relationship quality [21,22].

Given its role in social contexts, Itskovich and colleagues have proposed the oxytocin placebo hypothesis [23]. Oxytocin could affect various brain regions involved in pain perception and modulation, such as the periaqueductal gray (PAG) [24,25] and reward areas such as the nucleus accumbens and ventral tegmental area [25,26,27], reinforcing a positive expectation of pain relief. Oxytocin also impacts other brain areas involved in pain modulation, such as the amygdala and the prefrontal cortex [26,28], and has shown analgesic properties when administrated exogenously [29]. Lower endogenous oxytocin levels were linked to chronic pain in [30,31], while increases in endogenous oxytocin levels after non-pharmacological analgesic treatments were correlated with pain alleviation [32,33]. Current published research on oxytocin and placebo analgesia has mostly focused on the effects of *exogenous* oxytocin administration [34,35,36], i.e., the role of external oxytocin administration on healthy volunteers’ responses to a placebo, using experimentally induced pain. The results of these studies have been mixed, potentially because of differences in dosing, timing, participant sex, or pain paradigms [34]. However, there is currently no evidence for *endogenous* oxytocin’s role in responsiveness to placebos among patient populations.

Both the placebo response and changes in oxytocin secretion might be modulated by biological sex [35,36,37] but in a different manner. Given oxytocin’s role in labor and breast feeding, it was suggested that sex hormones might modulate oxytocin receptor sensitivity and differentiate its distributions [38]. In line with this, females and males tend to respond differently to exogenous oxytocin administration [35], and demonstrate different patterns of change in endogenous oxytocin levels [39]. Regarding the role of biological sex in the placebo response, some studies have demonstrated a higher placebo response in females [40,41], whereas others have shown no sex effects [42,43,44] or higher placebo responses in males [45,46]. These potential differences in the placebo effect between genders might be attributed to psychological constructs such as coping strategies, willingness to report pain, and hormonal fluctuations [40].

This study aimed to determine whether changes in endogenous oxytocin levels are associated with the placebo response and whether biological sex plays a role in patients with non-specific chronic back pain who receive a placebo injection. Our hypothesis was that increments in oxytocin will be associated with a higher placebo response. Regarding the role of sex, given the mixed results, we did not commit to a hypothesis but aimed to determine its effect.

## 2. Materials and Methods

This manuscript summarizes the results that were collected as part of a larger project aimed at assessing physiological, cognitive, and personal factors associated with the placebo response. The main results, including the prediction of placebo responses by the within-subject variability in pain intensity reports, were recently published elsewhere [47]. The larger study included a single study visit, in which participants completed questionnaires as well as physiological and neurophysiological assessments, and underwent quantitative sensory testing. A detailed description of the study timeline (Figure S1) and information about all assessments can be found in the Supplementary Materials. None of the other measures demonstrated significant results, either as standalone predictors of the placebo response, as additional independent variables, or as moderators of the results of this report; hence, they were not included in the manuscript. Given that the assessment of oxytocin was not the main purpose of this study, the analysis should be regarded exploratory, and in accordance, the results should be regarded as hypothesis generators.

### 2.1. Participants

Patients with chronic back pain treated at the Spine Unit of the Galilee Medical Center, Nahariya, Israel, were invited to participate in this study. Additional participants were recruited through social media and advertisements in the local press. Since participants were recruited using a convenience sample, we only have demographic data on those who agreed to take part.

The inclusion criteria included an age of between 18 and 80 years, non-specific back pain for 3 months or more, self-reported mean pain intensity of 3 or more on a numerical rating scale of 0 to 10 (NRS), and the ability to understand and read Hebrew. Adults up to 80 years old were included to capture the full range of patients in clinical practice, acknowledging that age-related changes in pain pathways might influence pain perception. The exclusion criteria included malignancy-related pain and pregnancy or breastfeeding.

### 2.2. Study Design

The study was designed as a sham randomized controlled trial (RCT) to mimic a randomized prospective double-blinded clinical trial, while in reality it was a case series with placebo deception (level 4 evidence).

To achieve a placebo response, the patients were told they would participate in a study evaluating the analgesic effect of “a magnetized vs. non-magnetized physiological isotonic solution injection”. This deceptive approach was chosen in accordance with local ethical guidance from the Declaration of Helsinki, which advises against deceiving participants about the treatment itself but allows deception regarding the study’s objective, if deemed necessary. The patients were randomized to alternately receive a magnetized or non-magnetized injection according to their consecutive order of participation. The solution used was drawn under aseptic conditions by a registered nurse from a single-use flask either pre-exposed for 24 h to a weak magnetic sheet (similar to a refrigerator magnet), or not. To comply with the sham study design, the magnetization of the solution was performed by one of the authors, while the injection was performed by another, who were both blinded, like the participants, to the allocation. All participants were instructed to maintain their regular medication treatment, including analgesics.

All study procedures were performed by the same study nurse for all participants.

### 2.3. Placebo Manipulation

Participants were given a subcutaneous injection of 0.5 mL 0.9% sodium chloride solution (saline) in the external upper buttock quadrant on the most painful side. An injection was chosen to support a robust placebo response. Additionally, the participants received verbal information about the documented analgesic effects observed in previous studies that used a similar procedure [48] and were informed that injections of physiological isotonic solutions have been shown to reduce labor pain. [49]. Specifically, the study nurse told participants, ‘You will receive an injection of a physiological isotonic solution to help relieve your back pain. There is a 50% chance that the solution is magnetized. Previous studies have shown that even non-magnetized solutions produce a noticeable pain-relieving effect. The analgesic effect of the injection is expected to peak within 30 min.”

The specifics about the solution’s properties (or rather, lack of) or use of the term “placebo” when explaining the treatment were not disclosed.

### 2.4. Pain Assessment and Calculation of Placebo Response

Current pain intensity was self-documented by all participants (both women and men) at two time points: at baseline and 30 min after placebo administration. A computerized visual analog scale (Co-VAS) was used, ranging from 0 to 100 (0 = ‘no pain’ and 100 = ‘worst imaginable pain’). Participants were instructed to drag a virtual cursor along the scale to the point that best represented their current back-pain intensity. The VAS is a widely used and validated instrument for assessing pain intensity, with good reliability and sensitivity in detecting clinically meaningful changes [50]. No significant differences were found between paper-based and computerized versions of the VAS [51].

The placebo response was calculated as the difference between pain intensity reported at the two time points of assessment (at baseline and 30 min after injection).

### 2.5. Saliva Collection and Oxytocin Measurement

The passive drool collection method (SalivaBio Collection Aid, Salimetrics, PA, USA) was employed for obtaining saliva samples for oxytocin measurement, which enables the collection of up to 1.8 mL of saliva. To ensure sample quality, participants were instructed to refrain from eating for one hour prior to the study visit and were provided with bottled water and instructed to drink in order to support salivary secretion. After centrifugation, the clarified samples were stored at −20 °C until further processing, which was performed after completion of data collection. Oxytocin levels in the saliva samples were measured with an oxytocin Enzyme-Linked Immuno-Sorbent Assay (ELISA) kit (Enzo Life Sciences, Farmingdale, NY, USA). A microplate reader (Varioskan LUX, Thermo Scientific, Waltham, MA, USA) was used to measure the optical density. Oxytocin concentration was determined by comparing the sample’s optical density to a standard curve. The change in oxytocin was calculated as the hormone level measured 30 min after the placebo injection minus the baseline oxytocin level. A dichotomous pattern of change (increase or decrease) was determined to address the heterogeneity in absolute values, as previously suggested [33,52].

### 2.6. Course of the Study Visit

Upon arrival at the study visit venue, participants were briefed about the study procedures and the planned injection, and they completed questionnaires. A self-reported initial VAS rating and a baseline salivary oxytocin sample were collected. The study nurse assisted the participants with the questionnaire and saliva collection, and was instructed to show empathy and kindness, and to positively interact with participants, to support the placebo response.

One hour after the saliva collection, the non-magnetized or magnetized saline injection was administered, accompanied by the verbal information. After placebo administration, participants waited for 30 min, and salivary oxytocin samples and pain VASs were collected for a second time. A detailed description of all study procedures can be found in the Supplementary Materials and in Figure S1. Upon completion of the visit, participants received an envelope containing 400 New Israeli Shekels and a gift-wrapped chocolate bar as compensation for participation.

### 2.7. Consent to Participate

At the beginning of the study visit, after being briefed about the study procedures and their right to withdraw at any time, participants signed an informed consent form.

### 2.8. Ethical Considerations

All procedures received regulatory approval from the ethical committees of the Galilee Medical Center (0044-20-NHR) and the University of Haifa (240/21). This study was part of clinicaltrials.gov protocol number NCT05994118. Data collection was performed between March 2021 and July 2023. Given the deceptive design, a few weeks after study completion, the participants were contacted, and the true aim of the study was disclosed. Although the participants were given the option to retract their data, none of them opted to exclude their data from the analyses.

### 2.9. Statistical Analyses

All statistical analyses used the SPSS Statistical Package for the Social Sciences software (IBM SPSS Statistics, version 27.0). Descriptive statistics were employed to evaluate the distributions of the sociodemographic and main study variables. Changes in variables of interest were calculated by subtracting the pre-measurement values from the post-measurement values; hence, positive changes reflect increases in the measurements, and negative values represent reductions. Normality was assessed using the Shapiro–Wilk and Kolmogorov–Smirnov tests, revealing that changes in oxytocin were not normally distributed. For this reason, nonparametric tests were used, and the oxytocin values are presented as medians (quartiles 25; 75). A Wilcoxon signed-rank test was used to assess whether changes in oxytocin were significant. A paired t-test was used to assess whether pain intensity significantly changed after placebo administration.

An independent-samples t-test, a Wilcoxon signed-rank test for independent samples, and chi-square tests were used to examine whether the variables mentioned above differed between the magnetized and the non-magnetized saline solution groups. Since no differences were found, the two study arms were combined into a single placebo group in all further analyses.

A Wilcoxon signed-rank test was used to assess if there were any sex-related differences in oxytocin levels.

Changes in oxytocin were then transformed into a dichotomous variable based on the pattern of response: increase or decrease. A chi-square analysis was used to assess the relationships between the oxytocin pattern and sex. A two-way analysis of variance (ANCOVA) with two independent variables (oxytocin pattern and sex) was conducted to explore the effect of oxytocin, sex, and the interaction of oxytocin and sex on the placebo response. The baseline oxytocin level was used as a co-variate.

The calculation of the number of participants needed was performed in the context of the larger study. For each main outcome measure, the number of participants needed was calculated using G*POWER (version 3.1.9.7), and a total of 130 participants was determined to be sufficient. Specifically for the case of oxytocin, based on RM-ANOVA with the following assumptions, 118 participants was deemed sufficient: effect size of 0.14, alpha of 0.05, power of 0.85, 2 groups and 2 measurements, and correlation among repeated measures of 0.5.

## 3. Results

**Figure 1:**
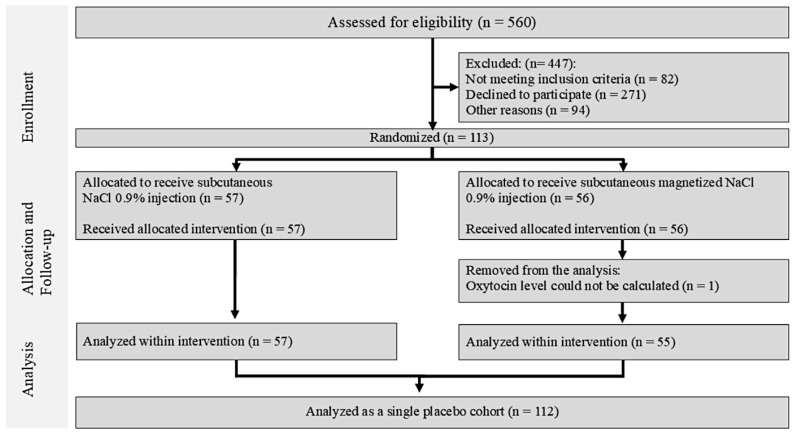
Flow diagram of the phases of the parallel randomized trial of the two study arms.

Recruitment started in March 2021 and was completed in July 2023. Among the 560 patients initially screened, 82 did not meet the inclusion criteria, 271 declined participation, and 94 patients could not be reached after initial contact, leaving 113 participants who successfully completed the study. A total of 56 of the 113 participants were assigned to the magnetized saline injection group, and 57 to the non-magnetized saline injection group. One participant was excluded from the magnetized saline injection group due to unreliable salivary oxytocin laboratory results (based on the ELISA test definitions). Eventually, 112 participants were included in the final analyses (Figure 1).

### 3.1. Sample Characteristics

Of the 112 participants who were analyzed, 50.9% were female. Their average age was 56.8 ± 15.3 years, with a range from 18 to 79 years. The majority (82%) reported experiencing back pain for at least a year. Three-quarters of the participants (75%) held either an academic degree or a professional diploma, and more than 80% were married or living with a partner. Their average body mass index, heart rate, and blood pressure fell within normal ranges. Table 1 and Table 2 summarize the participants’ demographic and medical information. No sex differences were observed in the demographic and clinical characteristics.

### 3.2. Pain Assessments and Change in Pain Intensity

**Figure 2:**
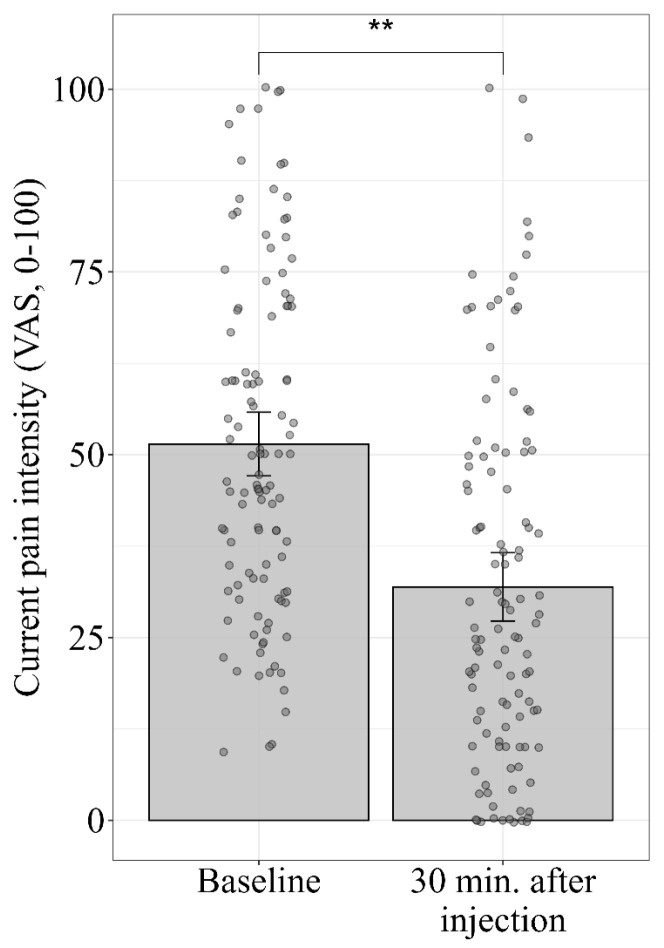
Average reported pain before and 30 min after placebo injection. The bars represent the average values of reported pain (VAS, 0–100) as assessed at baseline and 30 min following the placebo injection. Individual data points are presented in circles. The error bars represent the 95% confidence intervals (95%CI). ** *p*-value < 0.001.

The initial pain reported at baseline was 51.5 ± 23.3 (mean ± SD), and significantly declined by 19.5 ± 17.3 units to 31.9 ±2 5 (t_(111)_ = 11.9, *p* < 0.001) 30 min after the placebo injection, as presented in Table 3 and in Figure 2. The pain reduction, calculated as the difference between baseline and post-injection pain divided by the baseline pain, indicated a reduction of 37.8% in pain. This reduction is significant not only statistically, but also clinically, as the accepted Minimal Clinically Important Difference (MCID) is over 19/100 [53]. The properties of back pain as reported before and after the placebo manipulation, as well as the pain reduction (placebo response), are presented in Table 3.

### 3.3. Salivary Oxytocin Levels

Changes in oxytocin were distributed normally (in contrast to the baseline and post-injection values, which were not normally distributed), with a mean of change in oxytocin of −13.6 and with high variability (SD = 62.8). The distribution of changes in oxytocin is presented in Figure S2 (Supplementary Materials).

**Figure 3:**
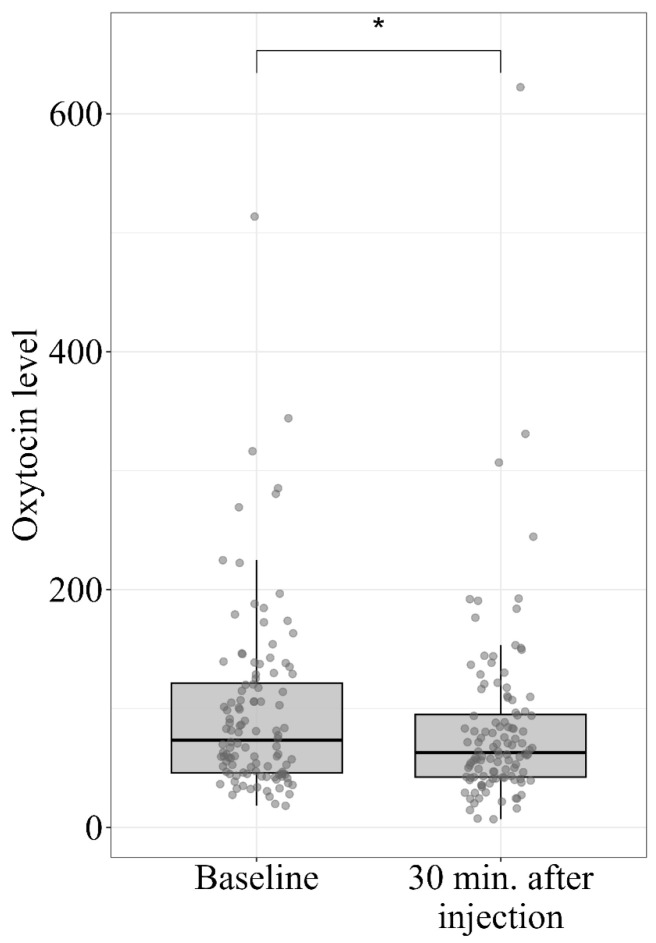
Oxytocin levels at baseline and 30 min after placebo injection. Oxytocin levels (pg/mL) as measured at baseline and 30 min following the placebo injection. Box-plots represent quartiles 25–75 of oxytocin values, and represent the median value. Individual data points are presented in circles. * *p*-value < 0.05.

The oxytocin levels are presented in Figure 3. The oxytocin levels showed a statistically significant decline (Z = −2.710, *p* = 0.007) in median values, which dropped from 73.3 to 62.9.

As expected, no significant differences were observed between the magnetized (*n* = 55) and non-magnetized (*n* = 57) injection groups across any of the variables mentioned earlier (Supplementary Tables S1–S4). Therefore, in all subsequent analyses, the two groups were combined and treated as a single placebo group.

A Wilcoxon signed-rank test for independent data assessing differences in changes in oxytocin between sexes showed a statistically significant difference (Z = −3.152, *p* = 0.002), with larger reductions in females (−12 [−49.9;−1.1]) than in males (1.2 [−21.9;21.3]) (Table 4).

Two patterns of changes in oxytocin (increase or decrease) were identified among participants, with 37.5% (N = 42) demonstrating increases in the hormone’s levels and 62.5% (N = 70) demonstrating decreases. Table 5 summarizes the oxytocin levels at baseline and after the placebo injection in the two subgroups based on the pattern of oxytocin change. Due to the differences found in baseline oxytocin levels between the two groups, in all further analyses, baseline oxytocin levels were used as covariates. In the supplementary materials, demographic and clinical variables, as well as pain and oxytocin measures, are compared between “oxytocin-increased” and “oxytocin-decreased” groups (Tables S5–S8), and between “placebo responders” and “non-responders” (Tables S9–S12). Due to high variability in oxytocin levels, an additional sensitivity analysis was performed, excluding outliers from the continuous variable representing change in oxytocin levels (Tables S13 and S14).

The transformed dichotomous variable was significantly associated with sex (χ^2^ = 10.7, df = 1, *p* = 0.001), indicating that females were more likely (77.2%) to show a decrease in oxytocin then an increase (22.8%) compared to males, who showed similar percentages of decreases (47.3%) and increases (52.7%) in oxytocin levels during the clinical visit. Importantly, the continuous variable of the change in oxytocin levels did not show a significant correlation with the placebo response (r = −0.068, *p* = 0.476). In addition, a linear regression with the placebo response as dependent variable and changes in oxytocin as the predictor, with or without the covariates age, gender, and menstrual cycle stage, yielded non-significant results (without covariates, *p* = 0.440; with covariates *p* = 0.237; none of the covariates were significant).

**Figure 4:**
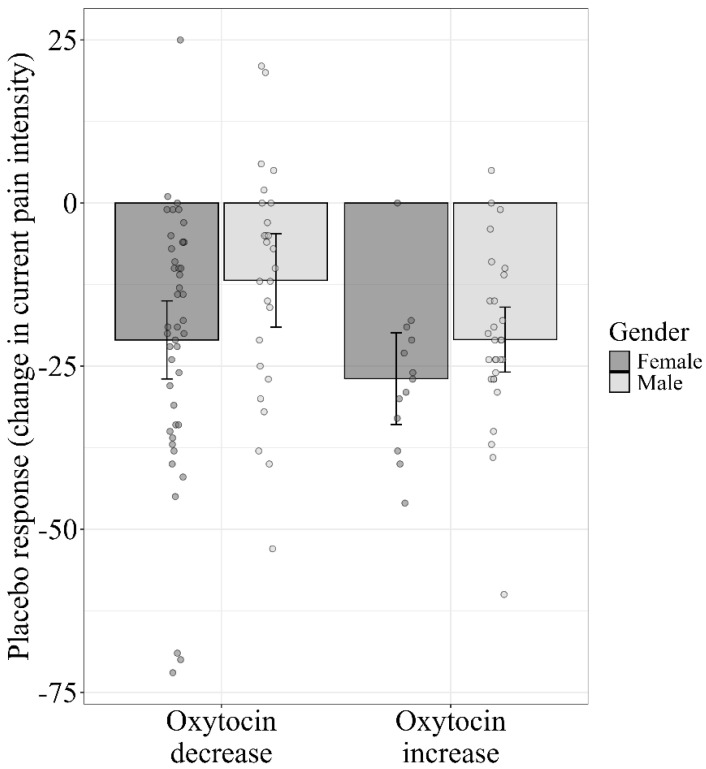
Effect of oxytocin change patterns and sex on the placebo response. The placebo response was higher among females, regardless of whether oxytocin increased or decreased after the placebo was injected (main effect of sex). The placebo response was also higher in the group whose oxytocin increased after the administration of the placebo, regardless of sex (main effect of changes in oxytocin level). Individual data points are presented in circles, with the error bars representing the 95%CI.

A two-way ANCOVA revealed two main effects: the placebo response was significantly (*p* = 0.024) higher in the group that showed an increase in oxytocin levels (−22.8 ± 12.8) compared to the group that showed a decrease (−17.6 ± 19.4), and the differences were significant between sexes, with significantly (*p* = 0.040) higher placebo response in females (−22.3 ± 18.2) compared to males (−16.6 ± 15.9). However, no statistically significant interaction between the oxytocin change pattern and sex was found (*p* = 0.687) (Figure 4). In addition, the baseline oxytocin level (the covariate) was not statistically significant (0.199).

**Figure 5:**
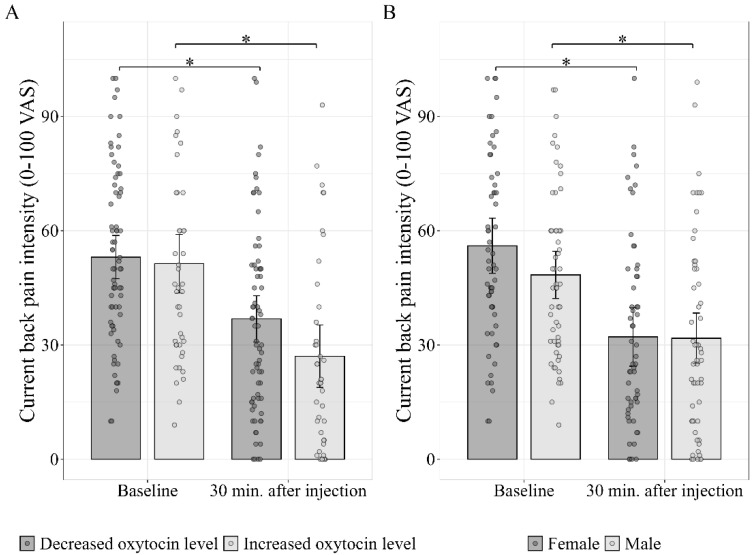
Interactions of time * oxytocin change pattern (**A**) and time * biological sex (**B**). Adjusted means of back pain intensity across time points, controlling for baseline oxytocin level. A repeated measures ANCOVA revealed a significant main effect of oxytocin change pattern (*F*_(1,111)_ = 5.250, *p* = 0.024, ηp^2^ = 0.047) (**A**). Similarly, a significant main effect of biological sex appeared (*F*(_1,111)_ = 4.311, *p* = 0.040, ηp^2^ = 0.039) (**B**). The baseline oxytocin level was a non-significant predictor (*F*_(1,111)_ = 1.667, *p* = 0.199, ηp^2^ = 0.015). The error bars represent the 95%CI. * *p*-value < 0.05.

We also ran a repeated measures ANCOVA (RM-ANCOVA), with baseline pain and pain post injection as within-subjects factors (‘time’); biological sex and oxytocin pattern of change as between-subjects factors; and baseline oxytocin levels as the covariate. There was a significant time * biological sex interaction (*F*_(1,111)_ = 4.31, *p* = 0.040, η^2^_p_ = 0.039) and a significant time * oxytocin pattern of change interaction (*F*_(1,111)_ = 5.25, *p* = 0.024, η^2^_p_ = 0.047). The covariate was non-significant (*p* = 0.199) (see Figure 5).

Since oxytocin levels might be affected by the menstrual cycle, we conducted the same analysis while adding participants with no menstruation—i.e., postmenopausal females (*n* = 40) and males (*n* = 55)—as a covariate. The effects of oxytocin pattern and sex on the placebo response were strengthened (*p* = 0.025 and *p* = 0.009, respectively). Finally, to assess if changes in oxytocin or gender moderate the prediction of the placebo response, we ran Hayes model 1. No significant moderation (*p* = 0.163) was found.

## 4. Discussion

The aim of this study was to assess if changes in oxytocin levels and biological sex are associated with the placebo response, as expressed by a reduction in self-reported pain intensity scores. Since we considered both magnetized and non-magnetized solutions to be identical, all participants from both study arms were evaluated as one placebo group. This was confirmed by our analysis of the demographic data, reported pain, and oxytocin levels, which did not differ between the magnetized and non-magnetized recipients.

We found that in non-specific chronic-back-pain patients whose endogenous oxytocin levels increased during the clinical visit, a higher placebo response was demonstrated compared to those whose oxytocin levels decreased. This finding is novel in several aspects. Previous published studies on the relationship between oxytocin and the analgesic placebo response yielded mixed results [54,55,56,57]. In addition, all previous studies were based on exogenous oxytocin administration and on experimental pain models, whereas our study focused on changes in endogenous oxytocin and on the clinical analgesic placebo response. Another novel aspect relates to our study population of chronic-back-pain patients. To the best of our knowledge, all previous studies on oxytocin and the analgesic placebo response were based on healthy volunteers [24,54,57,58]. The association observed in the current study between the oxytocin increase pattern and greater pain reduction is in line with the analgesic effect of oxytocin administration in clinical pain conditions [24,29,59].

Our findings provide partial support for the oxytocin placebo hypothesis proposed by Itskovich and colleagues [23]. According to this theory, affiliative interactions increase oxytocin levels. This, in turn, enhances the reward response [60,61], raising expectations of treatment efficacy and amplifying the placebo effect [62]. Oxytocin release is also known to affect brain areas involved in opioid release, such as the PAG [24,25], and areas involved in pain modulation and emotional regulation, such as the prefrontal cortex, amygdala, and anterior cingulate cortex [26,28]. Nonetheless, the fact that the continuous measure of change in oxytocin did not predict the placebo response suggests that oxytocin’s role in the placebo response might be modest, relative to the well-known psychological, physiological, and genetic underlying mechanisms of the placebo response [12,63,64,65,66,67].

In the current study, endogenous oxytocin levels were reduced at the group level. This finding is unexpected, as we hypothesized that the therapeutic interaction would increase oxytocin levels, as observed after social interactions [20,24,68,69]. However, as previously reported [68], the pattern of endogenous oxytocin response after prosocial interactions is highly variable, with some studies demonstrating no change [70] or even a reduction [71] after a positive interaction. Another possible explanation for the group-level decline during the clinical visit may relate to the timing of oxytocin measurements relative to the therapeutic interaction. It is possible that the measurements did not align with peak oxytocin secretion. Previous studies show that salivary oxytocin levels rapidly increase after intranasal administration of oxytocin [72], peaking 15 min after administration and gradually decreasing over a few hours. Since our study measured endogenous oxytocin levels, the exact time point of the oxytocin peak is unknown. Baseline oxytocin levels were measured 60 min before the placebo injection (the reference time point), but approximately one hour after the start of the visit, marking the actual beginning of the social interaction. Although there are some recommendations regarding the timing for baseline oxytocin sampling [68], these are less relevant for the exploration of the relationship between endogenous oxytocin change and pain reduction/placebo analgesia. It is possible that our baseline measurements were taken after oxytocin levels had already reached their peak since the social interaction started one hour before baseline sampling. Ninety minutes later, when these levels were measured again, they would be expected to continue declining, hence the reduction we observed at the group level. In addition, during the study visit, after the collection of the first oxytocin sample, participants underwent psychophysical testing, including exposure to experimental pain. This exposure may also have contributed to the observed decrease in oxytocin levels, which are generally lower in individuals experiencing pain [38]. Taken together, these findings suggest that the relationship between endogenous oxytocin and placebo analgesia may be more complex than a unidirectional increase following therapeutic interaction. Rather than negating the oxytocin–placebo link, our results highlight the need to consider individual variability, contextual influences, and non-linear patterns of oxytocin response.

Our findings also revealed a sex-specific pattern in oxytocin reactivity: women showed a decrease from baseline, whereas men exhibited a slight increase. Notably, no sex differences were observed in absolute oxytocin levels at baseline or after placebo injection. Such divergences in the change dynamics, rather than the absolute levels, are consistent with evidence that oxytocin release and receptor sensitivity are modulated by sex hormones and may differ in timing and feedback regulation across sexes [38,73]. Lastly, the study nurse was a female, and this could potentially explain the differences in changes in oxytocin between sexes [69]. In future studies, it might be beneficial to align the timing of both the clinician–patient interaction and the administration of the placebo, and to measure oxytocin levels more than twice. These additional steps could help further characterize the role of endogenous oxytocin in the analgesic placebo response.

Females in our study exhibited a significantly higher placebo response than males. The role of biological sex in analgesic placebo response has produced mixed results in previous research, with some studies showing higher placebo responses in females [40,41], some studies finding greater responses in males [45,46], and others finding no differences between sexes [42,43,44]. One possible explanation for these mixed results might be the sex of the examiner. In the current study, the examiner was a female nurse. As others have found, the interaction with a female might have affected both oxytocin secretion [74] and pain reports [75] differently in males and females. Other possible explanations include gender-specific psychosocial and physiological factors, such as coping strategies, willingness to report pain, and hormonal fluctuations [40]. These aspects warrant further investigation in future research.

In the current study, both statistically and clinically, a significant placebo response was observed, with a decrease of 37.8% in reported back-pain intensity 30 min after the administration of the placebo injection [53]. To assess the durability of the placebo effect over longer follow-up periods, further research is needed.

While much research has demonstrated the analgesic potential of the placebo response, most of the evidence originated from experimental placebo paradigms, utilized in both healthy participants and chronic pain patients [76,77,78,79,80]. There is less evidence based on changes in clinical pain after placebo administration (i.e., clinical placebo response), as assessed in this study. The magnitude of the placebo response in the current study is substantial and comparable to that reported in previously published papers of analgesia induced by placebo injection in chronic-back-pain patients [47,81]. The higher placebo response may be attributed to the invasive nature of the placebo administration method used in our study, which may be perceived by participants as more potent [82,83].

A few limitations deserve consideration. Since chronic back pain and pain alleviation are multifaceted, other factors, such as stress, expectations, and personality traits, could have influenced both the placebo response and oxytocin levels. We did not include an empty control arm in which pain was assessed before and after no treatment. Hence, we cannot differentiate between the placebo response and the placebo effect. Future studies should assess the relevance of oxytocin levels to the placebo effect. The exact study design and statistical power were established to achieve the main objective of the larger study, which was not focused on the role of oxytocin in the placebo response. Based on the power calculation, it might be that our sample was not large enough to identify a small effect size. As described, it might be that other study procedures affected the changes in oxytocin. In addition, peripheral oxytocin levels are only a proxy measure for central oxytocin levels. Hence, the oxytocin-related findings should be interpreted with caution. It should be noted, however, that salivary oxytocin is considered a more reliable peripheral measure than other peripheral methods, such as plasma or urinary oxytocin levels [84]. The timing of this study’s sessions, which were held in the morning or at noon, possibly affected oxytocin levels due to circadian differences. Additional menstrual differences may also have affected the results and should be considered in further research. Furthermore, the significant results of the current study are based on the transformed dichotomous oxytocin variable, whereas analyses using the continuous variable were non-significant. This implies that the findings in the current study are not robust, and future studies should further test the role of oxytocin in placebo analgesia.

The group-level decrease in oxytocin levels, and the absence of significant correlation with the continuous oxytocin variable, further highlight the caution needed in interpreting the findings. Given the exploratory nature of this report, alongside the above, the results of this study should be regarded as hypothesis generators and warrant replication in future prospective studies.

Lastly, in our study the nature of the social interaction between the study nurse and the participants, participants’ expectations that the treatment would be beneficial, and their perceptions regarding the credibility of the intervention were not assessed. These uncontrolled variables may have contributed to variability in both the placebo response and endogenous oxytocin secretion and should be addressed in future studies.

To conclude, this study highlights the role of endogenous oxytocin levels in the analgesic placebo response. It provides initial exploratory support for the oxytocin placebo hypothesis in a clinical cohort of chronic-back-pain patients. Better understanding of the underlying mechanisms of the analgesic placebo response could help with the development of new treatment approaches. Our results might also be relevant for other non-pain-related placebo responses.

## Figures and Tables

**Table 1:** Sociodemographic data of participants (N = 112).

Characteristics	Mean (SD)	Min–Max
**Age**	56.8 (15.4)	18–79
**Sex**	**Frequency, N (%)**	
Male	55 (49.1)	
Female	57 (50.9)	
Pre-menopause (% of the entire cohort)	17 (15.2)	
Post menopause (% of the entire cohort)	40 (35.7)	
**Education**		
Elementary school	4 (3.6)	
High school	24 (21.4)	
Secondary school/diploma	37 (33.0)	
Academic degree	47 (42.0)	
**Marital status**		
Unmarried	28 (25.0)	
Married/living with a partner	84 (75.0)	
**Employment**		
Yes	65 (58.0)	
No	47 (42.0)	

**Table 2:** Clinical data of participants (N = 112).

Characteristics	Mean (SD)	Min–Max
Body mass index	27.3 (5.2)	17.8–42.9
Baseline pulse (beats per minute)	70.8 (9.0)	48.1–91.9
Baseline systolic blood pressure (mm Hg)	120.8 (16.4)	85–160
Baseline diastolic blood pressure (mm Hg)	70.0 (10.6)	40–90
Duration of pain	**Frequency, N (%)**	
Less than 6 months	11 (9.7)	
6 to 12 months	8 (7.1)	
>12 months to 5 years	39 (34.5)	
>5 years	54 (48.7)	

**Table 3:** Back-pain intensity and the placebo response.

Time Point of Assessment	Mean ± SD	Minimum	Maximum
Baseline	51.5 ± 23.2	9.0	100.0
After placebo injection	31.9 ± 25.0	0.0	100.0
Pain delta (placebo response)	1–9.5 ± 17.3	7–2.0	25.0

**Table 4:** Sex-based differences in oxytocin levels.

		Median Oxytocin Level (Quartiles 25, 75)	Z	*p*
**Baseline**	Female	83.7 (46.0, 134.1)	−1.085	0.278
	Male	61.6 (45.1, 114.1)		
**30 min** **after injection**	Female	57.2 (40.4, 88.4)	−1.545	0.122
	Male	69.3 (46.8, 109.1)		
**Change**	Female	−12 (−49.9, −1.1)	−3.152	0.002
	Male	1.2 (−21.9, 21.3)		

**Table 5:** Oxytocin levels and changes by pattern of oxytocin change.

	Participants Exhibiting a Decrease in Oxytocin Levels (N = 70)	Participants Exhibiting an Increase in Oxytocin Levels (N = 42)	
	Median Oxytocin Level (Quartiles 25, 75)	Median Oxytocin Level (Quartiles 25, 75)	*p*
Baseline	84.7 (51.3, 129.3)	59.2 (41.2, 108.0)	0.032
30 min after injection	56.5 (39.1, 83.2)	80.6 (57.9, 145.7)	<0.001
Change	−22.2 (−49.5, −8.4)	20.9 (7.9, 31.1)	<0.001

## Data Availability

The original contributions presented in this study are included in the article. Further inquiries can be directed to the corresponding author.

## Supplementary Materials

The following supporting information can be downloaded at: https://www.mdpi.com/article/10.3390/jcm14207348/s1, Figure S1. timeline of the study visit; Figure S2. the distribution of changes in oxytocin; Table S1. Continuous demographic and medical characteristics by injection group; Table S2. Categorical/ordinal demographic and medical characteristics by injection group; Table S3. Summary of pain intensity and calculated placebo response, by injection groups; Table S4. Summary of the salivary oxytocin levels and change in oxytocin by injection groups; Table S5. Continuous demographic and medical characteristics by the pattern of change in oxytocin; Table S6. Categorical/ordinal demographic and medical characteristics by the pattern of change in oxytocin; Table S7. Summary of pain intensity and calculated placebo response, by the pattern of change in oxytocin; Table S8. Summary of the salivary oxytocin levels and change in oxytocin by the pattern of change in oxytocin; Table S9. continuous demographic and medical characteristics by placebo responders and non-responders; Table S10. Categorical/ordinal demographic and medical characteristics by placebo responders and non-responders; Table S11. Summary of pain intensity and calculated placebo response, by placebo responders and non-responders; Table S12. Summary of the salivary oxytocin levels and change in oxytocin by placebo responders and non-responders; Table S13. sex based differences in oxytocin levels, without outliers; Table S14. Salivary oxytocin levels and change in oxytocin by pattern of oxytocin change. References [85,86,87,88,89,90,91,92,93,94,95,96,97,98,99,100,101,102] are cited in Supplementary Materials.
