# Supplementary material for: The Role of Oxytocin and Sex in Analgesic Placebo-Response: Exploratory Analysis from a Sham Randomized Clinical Trial in Chronic Back-Pain Patients
Source: J Clin Med. 2025 Oct 17;14(20):7348. doi: 10.3390/jcm14207348 (PMC12564996; doi:10.3390/jcm14207348)
Supplement: Supplementary file 1 [file jcm-14-07348-s001.zip › jcm-3773043-supplementary.pdf]

Supplementary material:

This study is a part of a large PhD project. The 'Supplementary Methods' section presents various assessments and testing methods used in the broader project but not included in the current manuscript. In addition, the 'Supplementary Results' section presents comparisons of demographic data, reported pain intensities, and salivary oxytocin levels between recipients of magnetized and non-magnetized saline.

Supplementary methods:

Figure-S1: timeline of the study visit

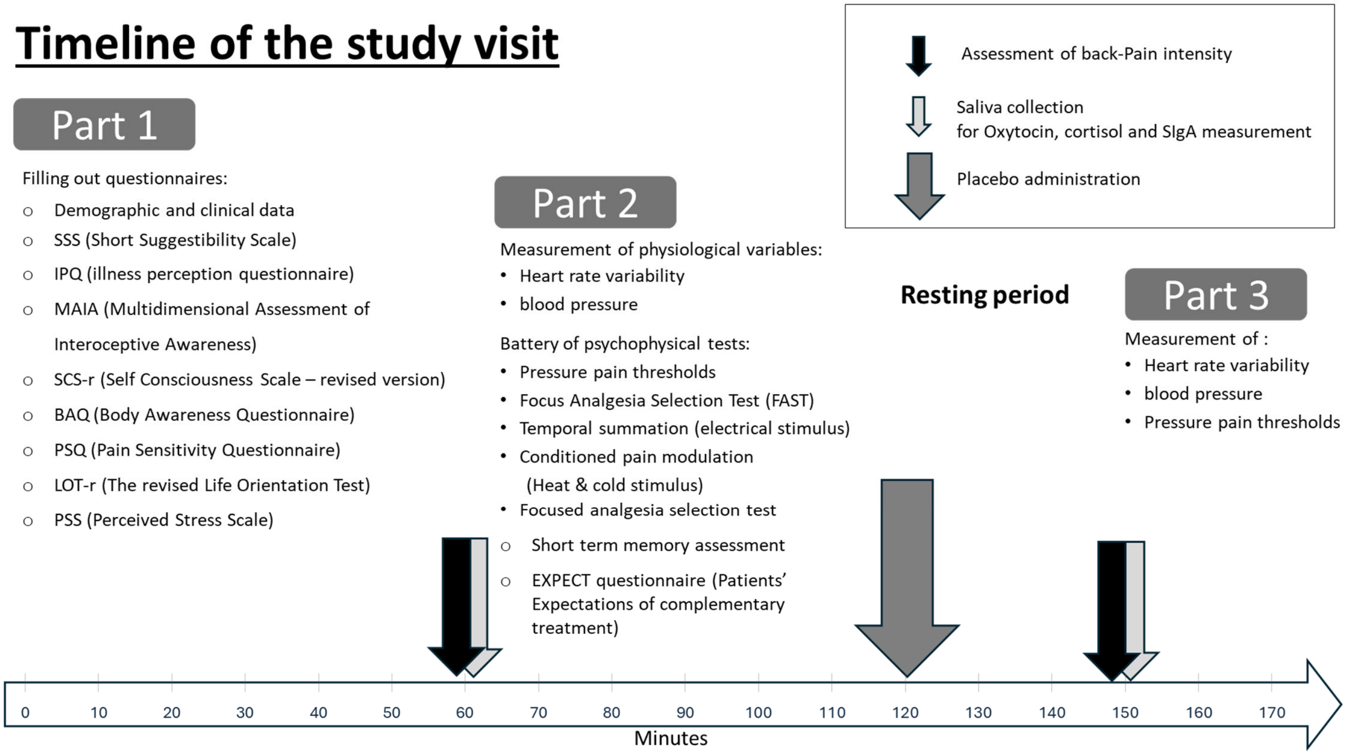

Details of the questionnaires utilized in the main project:

The **short suggestibility scale (SSS)** questionnaire is composed of 21 items drawn from the five subscales of the Multidimensional Iowa Suggestibility Scale (MISS) [86]. Participants were asked to indicate to what extent each of the items applies to him/her on a Likert scale (1=not at all or very slightly; 2=a little; 3=somewhat; 4=quite a bit; 5=a lot). The final score (range 21-105) is calculated as the sum of the 21-item scores. Higher score represents higher suggestibility level. The scale reliability as reported by the authors is 0.89 Cronbach's alpha.

The **Revised Illness Perception Questionnaire (IPQ-R)** (Moss-Morris et al., 2002), includes separate dimensions representing different aspects from which the cognitive perception of illness or health threat is composed. Each dimension consists of several items rated on a 1-5 Likert scale. Each dimension has a separate internal validity and can be used as a separate tool. The internal validity of all dimensions is high and ranges from 0.81 to 0.89 according to Cronbach's alpha.

BAQ (Body Awareness Questionnaire) includes 18 items in four subgroups (changes in body process, sleep and wake cycle, assessment of disease onset, and evaluation of body reactions). The BAQ aims to determine the level of body awareness. [87]. The reliability of the BAQ questionnaire is 0.88 Alpha Cronbach [88]

MAIA (Multidimensional Assessment of Interoceptive Awareness) questionnaire [89] is a 32-question questionnaire that examines the focus of attention and the subject's sense of the physiological state of his/her body with 8 sub-topics which are scored separately. The validity and reliability reported by the authors was strong-to-moderate and ranged between 0.66-0.87 Cronbach alpha for the different sub-topics.

The **Revised Self-Consciousness Scale questionnaire (SCS-R)** examines the self-awareness of the subject by assessing three key aspects of self-awareness: private self-awareness , public self-awareness and social anxiety (Scheier & Carver, 1985). The questionnaire contains 22 self-report items using a Likert scale ranging from 0 (“not like me at all”) to 3 (“very much like me”). The Cronbach alpha for these subscales calculated as 0.84, 0.75 and 0.79 respectively.

The **Pain Sensitivity Questionnaire (PSQ)** [91] contains 17 questions regarding common conditions in everyday life that may evoke pain. The overall score is calculated on a total PSQ scale. The authors reported a reliability of 0.91 according to Cronbach's alpha.

The revised version of the **Life Orientation Test- (LOT-R)** (Scheier, Carver, & Bridges, 1994) translated to Hebrew and validated by Drory (1997) includes six statements. Three express an optimistic approach and the other three a pessimistic approach. The scores in this questionnaire range from 6 to 30 and the higher the final score, the higher the level of optimism, and vice versa. The authors report a reliability of 0.80 according to Cronbach's alpha.

Perceived Stress Scale (PSS-10) [93] contains 10 statements describe a positive and negative mood. The reliability of PSS-10 range between 0.78-0.90 [94]

EXPECT questionnaire (short version) includes four items. Three items relate to the respondent's realistic expectations from the treatment, and one item relates to his/her hopes and wishes regarding the treatment. The intent of the question about hope is to increase respondents' focus on answering the 3 expectations questions in terms of their realistic expectations as opposed to their hopes for treatment outcomes. Alpha Cronbach for the short form of the EXPECT questionnaire is 0.89 [95].

Psychophysical tests performed:

Pain thresholds were evaluated by pressure pain model utilizing both manual algometer, and cuff pressure of the sphygmomanometer (Durga et al.,2016).

Temporal summation (TS) - was assessed using electrical stimulation generated by Digitimer DS7A (Digitimer Ltd, WelWyn Garden City, England). First, the electrical pain threshold was determined, and subsequently, ten consecutive electrical stimuli with one second interval between them at an intensity of 30% above the threshold were delivered. The participants rated their pain intensity on a numerical pain scale (NPS 0-100) after the first stimulus and after the last stimulus. TS magnitude was calculated as last-reported pain score minus first-reported pain score.

Conditioned pain modulation (CPM): CPM protocol consists of a painful test-stimulus given first as a standalone, and later in parallel with a different painful stimulus in a distant organ determined the conditioned stimulus. The CPM effect is calculated as the change in average pain rating between the stand alone test stimulus and the test stimulus under conditioned stimulus (Yarnitsky, 2015).

the 'test stimulus' was applied by a contact-heat Thermal Sensory Analyzer (2001 system, Medoc, Ramat-Yishai, Israel) with a 30x30mm Peltier surface stimulator, attached to the volar surface of the forearm of the participant dominant hand, delivering contact heat for_30s. The baseline temperature of the test stimulus was set to 32°C. Temperature increasing and decreasing rates were set to 1.0°C /second and 8.0°C/second, respectively. The 'conditioned stimulus' consisted of immersion of the participant's non-dominant hand into a cold-water (8-10°C) bath in a still position with the fingers wide apart.

CPM assessment –After a short training session each participant was exposed to 3 short contact-heat stimuli (43°C, 45°C, and 47°C) of 7 seconds plateau duration. Participants were asked to report on NRS 0-100, the intensity of pain induced by each stimulus. During this session, the intensity of the test stimulus was determined for each individual as the temperature that induces a pain experience at a magnitude of 50 on the NRS 0-100, termed the pain-50 intensity.

After the familiarization, the test was applied as mentioned above, starting with a standalone test stimulus, and continuing with the same procedure accompanied by conditioned stimulus.

Throughout the administration of the test stimulus, the participant was asked to report his/her pain intensity by NRS (0-100) at 3 time points: 10, 20, and 30 seconds after the temperature reaches the pain-50 intensity. During the conditioned stimulus, NRSs for the test stimulus were collected again at 40, 50, and 60 seconds after its initiation.

The focused analgesia selection test (FAST) was developed to identify degree of fluctuation in pain reporting [97]. In the FAST procedure, several thermal noxious stimuli of

varying intensities will be delivered to the ventral surface of the non-dominant arm (Medoc® Thermal Sensory Analyzer II with a thermode size of 30 × 30 mm). After a short training session, patients were asked to rate the pain intensity of each stimulus on a 0–100 numerical pain score (NPS 0-100) ranging from 0 (‘no pain’) to 100 (‘the worst pain imaginable’). The temperature raised from a baseline of 32°C, peak for 3 seconds at one out of seven predetermined temperatures (43, 45, 46, 47, 48, 49, or 50°C), with a total stimulus duration of 8 seconds (including ramping up and down). (Each temperature is presented seven times in a random block-ordered design with a total of 49 stimuli). Participants were reported their pain intensity following each stimulus. Stimuli is triggered automatically every 20 seconds. The location of the thermode is adjusted every 14 stimuli to minimize sensitization and/or habituation effects [97]. The total time of the FAST procedure is approximately 20 minutes. The main outcome measure of the FAST is the calculated R2 value of the relationship between stimulus intensities and pain reports. Secondary outcome measures include the coefficient of variance (CoV) and interclass correlation coefficient (ICC) of relationships between stimulus intensities and pain reports.

Short-term memory was assessed by digit-span-task. This is a subtest of the Wechsler intelligence assessment III for adults, based on a simple task in which the participant is asked to repeat a series of numbers presented to him verbally. The participant were asked to repeat the numbers - both in the order of presentation and in the reverse order. This short test allows for short-term memory function evaluation based on participants' performance (number of errors) [98], with good reliability ranging between 0.85-0.92 [99]

Supplementary results:

Tables S1-S4 summarize the results of analyses comparing the demographic data, the reported pain intensities, the placebo response and the salivary oxytocin levels between the magnetized and non-magnetized saline recipients.

Table S1. Continuous demographic and medical characteristics by injection group

**Characteristics**	**Saline injection N = 57 mean (SD)**	**Magnetized saline injection N = 56 mean (SD)**	***P***
Age	56.4(15.2)	57.2(15.6)	0.781
Body mass index	27.2(5.0)	27.4(5.4)	0.836
Baseline pulse rate	70.7(9.4)	70.9(8.6)	0.907
Baseline systolic blood pressure	119.4(15.7)	122.2(17.2)	0.371
Baseline diastolic blood pressure	68.7(10.2)	71.3(10.9)	0.198

Table S2. Categorical/ordinal demographic and medical characteristics by injection group

**Characteristics**	**Saline injection *N* = 57 *N* (%)**	**Magnetized saline injection N = 56 N (%)**	***P***
**Sex**			
Male	29.0(50.9)	26.0(47.3)	0.703
Female	28.0(49.1)	29.0(52.7)	
**Duration of pain**			
<6 months	6.0(10.7)	5.0(9.1)	0.805
6 to 12 months	5.0(8.9)	3.0(5.5)	
>12 months to 5 years	20.0(35.7)	18.0(32.7)	

>5 years	25.0(44.6)	29.0(52.7)	
**Education**			
Elementary school	4.0(7.0)	0.0(0.0)	
High school	14.0(24.6)	10.0(18.2)	0.158
Secondary school	18.0(31.6)	19.0(34.5)	
Academic degree	21.0(36.8)	26.0(47.3)	
**Marital status**			
Unmarried	14.0(50.0)	14.0(50.0)	0.913
Married/living with a partner	43.0(51.2)	41.0(48.8)	
**Currently employed**			
No	21.0(36.8)	26.0(47.3)	0.263
Yes	36.0(63.2)	29.0(52.7)	

Table S3. Summary of pain intensity and calculated placebo response, by injection groups

	**Saline injection N = 57 Mean (SD)**	**Magnetized saline injection N = 56 Mean (SD)**	***P***
**Pain before injection**	51.6(23.7)	51.3(22.9)	0.955
**Pain 30 minutes after injection**	32.1(23.2)	31.7(27.0)	0.937
**Placebo response**	-19.5(16.5)	-19.6(18.3)	0.969

Table S4. Summary of the salivary oxytocin levels and change in oxytocin by injection groups

	**Saline injection N = 57 Median (quartiles 25 , 75)**	**Magnetized saline injection N = 56**	***P***

		**Median (quartiles 25 , 75)**	
**Baseline Oxytocin**	59.9(44.9 , 127.2)	81.7(51.4 , 120.3)	0.318
**Oxytocin 30 minutes after injection**	62.0(39.8 , 95.4)	65.3(46.6 , 97.5)	0.347
**Change in Oxytocin**	-7.2(-27.9 , 18.5)	-5.8(-30.2 , 9.8)	0.942

Tables S5-S8 summarize the results of analyses comparing the demographic data, the reported pain intensities, the placebo response and the salivary oxytocin levels between the two groups based on oxytocin pattern of change (increased vs. decreased).

Table S5. Continuous demographic and medical characteristics by the pattern of change in oxytocin

**Characteristics**	**Oxytocin increased**<br>**N = 42**<br>**mean (SD)**	**Oxytocin decreased**<br>**N = 70**<br>**mean (SD)**	***P***
Age	56.9(14.2)	56.7(16.1)	0.935
Body mass index	28.1(4.6)	26.8(5.5)	0.216
Baseline pulse rate	71.9(9.0)	70.2(9.0)	0.337
Baseline systolic blood pressure	120.7(14.6)	120.8(17.5)	0.982
Baseline diastolic blood pressure	71.0(12.2)	69.4(9.6)	0.443

Table S6. Categorical/ordinal demographic and medical characteristics by the pattern of change in oxytocin

**Characteristics**	**Oxytocin increased**<br>**N = 42**<br>***N* (%)**	**Oxytocin decreased**<br>**N = 70**<br>**N (%)**	***P***
**Sex**			
Male	29(69.0)	26(37.1)	0.001
Female	13(31.0)	44(62.9)	
**Duration of pain**			
<6 months	1(2.4)	10(14.3)	
6 to 12 months	1(2.4)	7(10)	0.010
>12 months to 5 years	16(39)	22(31.4)	
>5 years	23(56.1)	31(44.3)	
**Education**			0.232

Elementary school	1(2.4)	3(4.3)	
High school	7(16.7)	17(24.3)	
Secondary school	23(32.9)	23(32.9)	
Academic degree	20(47.6)	27(38.6)	
**Marital status**			
Unmarried	6(14.3)	22(31.4)	0.043
Married/living with a partner	36(85.7)	48(68.6)	
**Currently employed**			
No	16(38.1)	31(44.3)	0.520
Yes	26(61.9)	39(55.7)	

Note that the significant finding about sex is already discussed in the main article. Regarding the significant p-value in duration of pain – in both groups majority of participants reported duration of pain > 5 years. The difference that drive the significant results is in the <12 months. This is based on low number of participants and could be interpreted as either due to chance, or another interpretation is that those with lower duration of pain tend to show reduction in oxytocin. Lastly, marital status also found significant, with unmarried found more in the oxytocin decrease than in the oxytocin increase groups. This might suggest that those who are not sharing their life with significant other tend to show decrease in oxytocin.

Table S7. Summary of pain intensity and calculated placebo response, by the pattern of change in oxytocin

	**Oxytocin increased N = 42 Mean (SD)**	**Oxytocin decreased N = 70 Mean (SD)**	***P***
**Pain before injection**	48.4(23.7)	53.3(22.9)	0.277
**Pain 30 minutes after injection**	25.6(25.0)	35.7(24.5)	<0.037
**Placebo response**	-23.0(15.1)	-17.6(19.4)	<0.091

Note that this significant finding is already presented and discuss in the main article.

Table S8. Summary of the salivary oxytocin levels and change in oxytocin by the pattern of change in oxytocin

	**Oxytocin increased**	**Oxytocin decreased**	***P***

	N = 99<br>Median<br>(quartiles 25 , 75)	N = 13<br>Median<br>(quartiles 25 , 75)	
**Baseline Oxytocin**	59.2(41.2 , 108.0)	84.7(51.3 , 129.3)	0.032
**Oxytocin 30 minutes after injection**	80.6(57.9 , 145.7)	56.5(39.1 , 83.2)	<0.001
**Change in Oxytocin**	20.9(7.9 , 31.12)	-22.2(-49.5 , -8.4)	<0.001

Note that the significant p-values in table S8 are artifact, due to the fact that the groups were divided based on the change in oxytocin.

Tables S9-S12 summarize the results of analyses comparing the demographic data, the reported pain intensities, the placebo response and the salivary oxytocin levels between the two groups based on placebo responder status (i.e. demonstrated reduction in pain, or not, using "0" as cutoff).

Table S9. continuous demographic and medical characteristics by placebo responders and non-responders

Characteristics	Placebo responders N = 99 mean (SD)	Placebo non-responders N = 13 mean (SD)	*P*
Age	56.5(15.3)	58.4(16.2)	0.685
Body mass index	27.5(5.3)	25.7(3.7)	0.260
Baseline pulse rate	70.8(9.3)	71.1(6.3)	0.927
Baseline systolic blood pressure	120.5(16.2)	122.7(18.9)	0.654
Baseline diastolic blood pressure	69.9(10.3)	70.4(9.0)	0.877

Table S10. Categorical/ordinal demographic and medical characteristics by placebo responders and non-responders

Characteristics	Placebo responders N=99 *N* (%)	Placebo non-responders N = 13 N (%)	*P*
**Sex**			
Male	46(46.5)	9(69.2)	0.123
Female	53(53.5)	4(30.8)	
**Duration of pain**			
<6 months	7(7.1)	4(33.3)	0.321
6 to 12 months	8(8.1)	0(0)	

>12 months to 5 years	36(36.4)	2(16.7)	
>5 years	48(48.5)	6(50.0)	
**Education**			
Elementary school	4(4.0)	0(0)	
High school	20(20.2)	4(30.8)	0.931
Secondary school	34(34.3)	3(23.1)	
Academic degree	41(41.4)	6(46.2)	
**Marital status**			
Unmarried	26(26.3)	2(15.4)	0.394
Married/living with a partner	73(73.7)	11(84.6)	
**Currently employed**			
No	40(40.4)	7(53.8)	0.356
Yes	59(59.6)	6(46.2)	

Table S11. Summary of pain intensity and calculated placebo response, by placebo responders and non-responders

	**Placebo responders N = 99 Mean (SD)**	**Placebo non-responders N = 13 Mean (SD)**	***P***
**Pain before injection**	51.5(22.9)	51.1(26.5)	0.951
**Pain 30 minutes after injection**	28.5(23.1)	57.6(25.5)	<0.001
**Placebo response**	-23.0(15.1)	6.54(9.1)	<0.001

Note that the significant p-values in table S11 are artifact, due to the fact that the groups were divided based on the placebo response (responders vs. non-responders).

Table S12. Summary of the salivary oxytocin levels and change in oxytocin by placebo responders and non-responders

	**Placebo responders N = 99 Median (quartiles 25 , 75)**	**Placebo non-responders N = 13 Median (quartiles 25 , 75)**	***P***
**Baseline Oxytocin**	70.7(45.4 , 120.3)	85.7(46.3 , 139.2)	0.448
**Oxytocin 30 minutes after injection**	65.3(43.3 , 94.4)	43.2(39.6 , 102.8)	0.338
**Change in Oxytocin**	-7.4(-29.5 , 12.8)	-21.9(-40.4 , 9.5)	0.528

sensitivity analyses of salivary oxytocin levels without outliers

Due to high variability in oxytocin levels, outliers were excluded from the continuous variable representing change in oxytocin levels calculated as a oxytocin levels 30min. after injection minus oxytocin at baseline. Outliers were identified using the conventional interquartile range (IQR, IQR=Q3-Q1), using the rule Q1-1.5*IQR for lower end of outliers in the variable distribution and Q3+1.5*IQR for outliers on the upper end of the distribution.

For the delta change the IQR was 12.46-(-29.84) = 42.30. Then the application of the rule resulted in exclusion of every participant with values below -93.29 (-29.84-1.5*42.30) and above 75.92 (12.46+1.5*42.30). Accordingly, a total of 14 participants were considered outliers and the following sensitivity analyses were performed without those participants.

Table S13. sex based differences in oxytocin levels, without outliers

		**Median oxytocin level (quartiles 25, 75)**		**P**

**Baseline**	Female	69.5 (44.7, 104.5)	.227	0.820
	Male	60.7 (44.9, 106.3)		
**30 mins after injection**	Female	58.0 (41.0, 87.3)	.945	0.345
	Male	64.7 (43.0, 95.0)		
**Change**	Female	-10.1 (-25.7, 1.6)	2.359	0.018
	Male	1.0 (-21.1, 20.9)		

Oxytocin levels showed a statistically significant decline (Z = -2.183, p = 0.029) in median values, which dropped from 67.3 to 61.5.

Table S14. Salivary oxytocin levels and change in oxytocin by pattern of oxytocin change

	**Participants exhibiting a decrease in oxytocin levels (N = 70)**	**Participants exhibiting an increase in oxytocin levels (N = 42)**	
	**Median oxytocin level (quartiles 25 , 75)**	**Median oxytocin level (quartiles 25 , 75)**	***P***
Baseline	79.3(47.0 , 105.6)	59.2(39.6 , 105.8)	0.135

30 minutes after injection	55.7(38.3 , 73.9)	77.7(56.0 , 130.7)	<0.001
Change	-17.8(-35.3 , -7.5)	16.6(7.4 , 24.4)	<0.001

The transformed dichotomous variable was significantly associated with sex (χ^2 = 7.5, df = 1, P = 0.006), indicating that females were more likely (75.0%) to show a decrease in oxytocin then an increase (25.0%) compared to males, who showed similar percentages of decreases (48.0%) and increases (52.0%) in oxytocin levels during the clinical visit. Importantly, the continues variable change in oxytocin levels did not show significant correlation with the placebo response (r=-0.127, p=0.213)

A two-way ANCOVA revealed two main effects: the placebo-response was significantly (p = 0.008) larger in the group that showed an increase in oxytocin levels (-23.5 ± 12.9) compared to the group that showed a decrease (-16.5 ± 17.2); and between sexes, with significantly (p = 0.019) larger placebo-response in females (-22.0 ± 14.8) compared to males (-16.6 ± 16.7). However, no statistically significant interaction between the oxytocin change pattern and sex was found (p = 0.954) (Figure 4). In addition, baseline oxytocin level (the covariate) was not statistically significant (0.882).

We also run a Repeated Measures ANCOVA (RM-ANCOVA), with baseline pain and pain post injection as within-subjects' factors ('time'), with biological sex and oxytocin pattern of change as between subjects' factors, and baseline oxytocin levels as covariate. There was a significant time* biological sex interaction ($F_{(1, 93)}$ = 5.70, p= .019, η^2_p = .058) and a significant time* oxytocin pattern of change ($F_{(1, 93)}$ = 7.46, p= .008, η^2_p = .074). The covariate was non-significant (p=0.882)

Since oxytocin levels might be affected by the menstrual cycle, we conducted the same analysis while adding participants with no menstruation—i.e., postmenopausal females (n=35) and males (n=50)—as a covariate. The effects of the oxytocin pattern and sex on the placebo response were strengthened (p = 0.011 and p = 0.006, respectively)

Figure S2. the distribution of changes in oxytocin

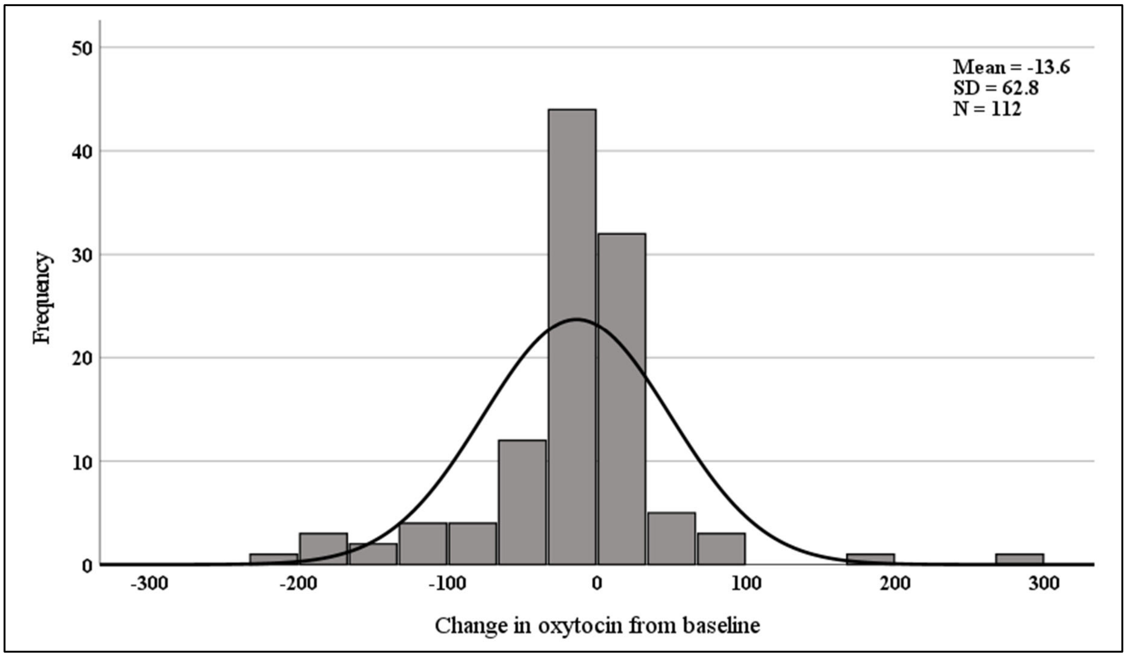

Supplementary References:

*(The attached list pertains only to citations of the material presented in the supplementary section and does not appear in the main article.)

1. Arendt-Nielsen L, Brennum J, Sindrup S, Bak P. (1994) Electrophysiological and psychophysical quantification of temporal summation in the human nociceptive system. *Eur J Appl Physiol Occup Physiol*. 68(3):266-273. https://doi.org/10.1007/BF00376776
2. Cohen, S., Kamarck, T., & Mermelstein, R. (1983). A global measure of perceived stress. *Journal of Health and Social Behavior*, *24*(4), 385–396. https://doi.org/10.2307/2136404
3. Durga, P., Wudaru, S. R., Khambam, S. K. R., Chandra, S. J., & Ramachandran, G. (2016). Validation of simple and inexpensive algometry using sphygmomanometer cuff and neuromuscular junction monitor with standardized laboratory algometer. *Journal of Anaesthesiology Clinical Pharmacology*, *32*(1), 74–79. https://doi.org/10.4103/0970-9185.173390
4. Jones, S. M. W., Lange, J., Turner, J., Cherkin, D., Ritenbaugh, C., Hsu, C., … Sherman, K. (2016). Development and Validation of the EXPECT Questionnaire: Assessing Patient Expectations of Outcomes of Complementary and Alternative Medicine Treatments for Chronic Pain. *Journal of Alternative and Complementary Medicine*, *22*(11), 936–946. https://doi.org/10.1089/acm.2016.0242
5. Kotov, I., Bellman, S. B., & Watson, D. B. (2004). *Multidimensional Iowa Suggestibility Scale (MISS)*. http://hdl.handle.net/1951/60894
6. Mehling, W. E., Price, C., Daubenmier, J. J., Acree, M., Bartmess, E., & Stewart, A. (2012). The Multidimensional Assessment of Interoceptive Awareness (MAIA). *PLoS ONE*, *7*(11), e48230. https://doi.org/10.1371/journal.pone.0048230
7. Moss-Morris, R., Weinman, J., Petrie, K., Horne, R., Cameron, L., & Buick, D. (2002). The Revised Illness Perception Questionnaire (IPQ-R). *Psychology & Health*, *17*(1), 1–16.

https://doi.org/10.1080/08870440290001494

8. Price DD, Hayes RL, Ruda M, Dubner R. (1978). Spatial and temporal transformations of input to spinothalamic tract neurons and their relation to somatic sensations. *J Neurophysiol*. 41(4):933-947. https://doi.org/10.1152/jn.1978.41.4.933
9. Ruscheweyh, R., Marziniak, M., Stumpenhorst, F., Reinholz, J., & Knecht, S. (2009). Pain sensitivity can be assessed by self-rating: Development and validation of the Pain Sensitivity Questionnaire. *Pain*, *146*(1–2), 65–74. https://doi.org/10.1016/j.pain.2009.06.020
10. Ryan, J. J., Arb, J. D., Paul, C. A., & Kreiner, D. S. (2000). Reliability of the WAIS-III subtests, indexes, and IQs in individuals with substance abuse disorders. *Assessment*, *7*(2), 151–156. https://doi.org/10.1177/107319110000700206
11. Scheier, Michael F., & Carver, C. S. (1985). The Self-Consciousness Scale: A Revised Version for Use with General Populations. *Journal of Applied Social Psychology*, *15*(8), 687–699. https://doi.org/10.1111/j.1559-1816.1985.tb02268.x
12. Scheier, Michael F., Carver, C. S., & Bridges, M. W. (1994). Distinguishing Optimism From Neuroticism (and Trait Anxiety, Self-Mastery, and Self-Esteem): A Reevaluation of the Life Orientation Test. *Journal of Personality and Social Psychology*, *67*(6), 1063–1078. https://doi.org/10.1037/0022-3514.67.6.1063
13. Shields, S. A., Mallory, M. E., & Simon, A. (1989). The Body Awareness Questionnaire: Reliability and Validity. *Journal of Personality Assessment*, *53*(4), 802–815. https://doi.org/10.1207/s15327752jpa5304_16
14. Taylor, J. M. (2015). Psychometric analysis of the ten-item perceived stress scale. *Psychological Assessment*, *27*(1), 90–101. https://doi.org/10.1037/a0038100
15. Treister, R., Eaton, T. A., Trudeau, J. J., Elder, H., & Katz, N. P. (2017). Development and preliminary validation of the focused analgesia selection test to identify accurate pain reporters. *Journal of Pain Research*, *10*, 319–326. https://doi.org/10.2147/JPR.S121455

16. Unal, A., Altug, F., Erden, A., Cavlak, U., & Senol, H. (2020). Validity and reliability of the Body Awareness Questionnaire in patients with non-specific chronic low back pain. *Acta Neurologica Belgica*. https://doi.org/10.1007/s13760-020-01399-y

17. Woods, D. L., Kishiyama, M. M., Yund, E. W., Herron, T. J., Edwards, B., Poliva, O., … Reed, B. (2011). Improving digit span assessment of short-term verbal memory. *Journal of Clinical and Experimental Neuropsychology*, *33*(1), 101–111. https://doi.org/10.1080/13803395.2010.493149

18. Yarnitsky, David. (2015, April 1). Role of endogenous pain modulation in chronic pain mechanisms and treatment. *Pain*, Vol. 156, pp. S24–S31. https://doi.org/10.1097/01.j.pain.0000460343.46847.58
